# Transrectal spectral Doppler reveals uterine hyperemia in Tharparkar cows with subclinical endometritis

**DOI:** 10.3389/fvets.2026.1736926

**Published:** 2026-04-07

**Authors:** Uttam Kumar Sahu, Brijesh Kumar, M. H. Khan, Mayank Singh, Chinmay Warghat, Vishnu Vadera, Amit Kumar, Athidi Lokavya Reddy, Nitish Singh Kharayat, Vandana, V. S. Chouhan, Piyush Rajput, Mahesh Kumar, Laxmi Sahu, Harideep Verma, Ghanshyam Sahu, S. K. Singh

**Affiliations:** 1 Division of Animal Reproduction, ICAR-Indian Veterinary Research Institute, Bareilly, Uttar Pradesh, India; 2 ICAR-Indian Institute of Agricultural Biotechnology, Ranchi, Jharkhand, India; 3 College of Veterinary Science, Korutla, P. V. Narsimha Rao Telangana Veterinary University, Hyderabad, Telangana, India; 4 Division of Temperate Animal Husbandry, ICAR-Indian Veterinary Research Institute, Muksteshwar Campus, Nainital, Uttarakhand, India; 5 ICAR-Indian Agricultural Research Institute (IARI), Hazaribagh, Jharkhand, India; 6 Division of Physiology and Climatology, ICAR-Indian Veterinary Research Institute, Bareilly, Uttar Pradesh, India; 7 Division of Veterinary Surgery and Radiology, ICAR-Indian Veterinary Research Institute, Bareilly, Uttar Pradesh, India; 8 Livestock Production and Management, College of Veterinary Science and Animal Husbandry, Anjora, Chhattisgarh, India; 9 Division of Extension Education, ICAR-Indian Veterinary Research Institute, Bareilly, Uttar Pradesh, India; 10 Division of Biochemistry, ICAR-Indian Veterinary Research Institute, Bareilly, Uttar Pradesh, India

**Keywords:** Doppler indices, pulsatility index, resistance index, subclinical endometritis, Tharparkar cattle

## Abstract

**Introduction:**

Subclinical endometritis (SCE) is a postpartum uterine disorder characterized by cytological inflammation without clinical signs, impairing fertility and productivity in dairy cattle. Diagnosis relies on endometrial cytology, which is invasive and impractical under field conditions. Doppler ultrasonography enables non-invasive assessment of uterine blood flow (UBF) and may detect inflammation-associated vascular changes. However, uterine hemodynamic characterization in indigenous *Bos indicus* breeds affected with SCE remains limited. This study evaluated Doppler-derived uterine perfusion dynamics in healthy and SCE-affected Tharparkar cows.

**Materials and methods:**

Twenty lactating multiparous Tharparkar cows (70 ± 3.34 days postpartum) were screened using endometrial cytology; cows with ≥5% polymorphonuclear cells were classified as SCE-positive (*n* = 10), and healthy cows served as controls (*n* = 10). Transrectal color and spectral Doppler examinations of the middle uterine artery (MUA) were performed every three days throughout one complete estrous cycle. Resistance index (RI), pulsatility index (PI), time-averaged maximum velocity (TAMV), blood flow volume (BFV), and MUA diameter were recorded. Serum progesterone (P_4_) was measured by ELISA. Data were analyzed using independent t-tests, repeated-measures ANOVA (GLM), and Pearson correlation.

**Results:**

SCE cows exhibited significantly lower RI and PI across multiple cycle days (*P* < 0.05), indicating increased uterine perfusion. TAMV, BFV, and MUA diameter were significantly higher in SCE cows (*P* < 0.05). RI positively correlated with P_4_ concentration (*r* = 0.77; *P* < 0.0001) and corpus luteum size (*r* = 0.64; *P* < 0.0001), while BFV showed positive correlations with TAMV (*r* = 0.82; *P* < 0.0001) and MUA diameter (*r* = 0.78; *P* < 0.0001), and negative correlations with RI (*r* = −0.57; *P* < 0.0001) and P_4_ (*r* = −0.59; *P* < 0.0001). Production traits had no significant influence on Doppler indices (*P* > 0.05).

**Conclusion:**

Subclinical endometritis in Tharparkar cows is associated with distinct uterine hemodynamic alterations characterized by increased blood flow and reduced Doppler resistance indices. These findings establish baseline Doppler reference patterns for an indigenous *Bos indicus* breed and support the potential utility of Doppler ultrasonography as a non-invasive adjunct tool for detecting uterine inflammation. Further studies integrating vascular and biochemical markers are warranted to enhance diagnostic precision.

## Graphical abstract

**Figure:**
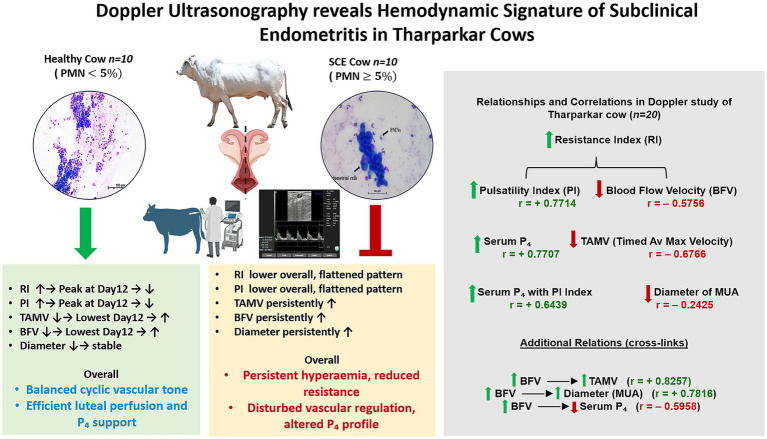

## Introduction

Indigenous *Bos indicus* cattle are central to rural livelihoods in many low- and middle-income countries, providing milk, draft power and income. Although *Bos indicus* breeds are relatively heat tolerant (1), tropical environments can increase exposure to reproductive pathogens and the prevalence of uterine infections (2, 3). Reported prevalence of postpartum uterine infection in cattle varies widely (12.7–47.9%), with metritis (8–40%), clinical endometritis (5–30%) and subclinical endometritis (SCE; reported 11–70%) among the most common (4, 5). Subclinical endometritis, also known as cytological endometritis, is defined as endometrial inflammation detected on cytology in the absence of clinical signs or systemic illness (6). Uterine diseases, especially clinical endometritis and SCE, have been found to decrease milk production in cows by 0.6–1.03 kg/day, reduce milk fat and protein, and increase somatic cell counts in milk (7, 8) leading to losses of billions of dollars annually global dairy industry (9). Unlike clinical infections, SCE is asymptomatic and difficult to detect, with diagnosis largely reliant on endometrial cytology. However, this method is invasive, labor-intensive, and poorly standardized, limiting its utility under field conditions. B-mode ultrasound can detect uterine fluid and wall thickening but has limited sensitivity and specificity for SCE; hence non-invasive physiological markers such as Doppler indices may be useful adjuncts (10). Inflammation is typically accompanied by vasodilation and increased tissue perfusion mediated by agents such as histamines (11), bradykinins (12), prostaglandins (13), and nitric oxide (14). During uterine infection, there is release of inflammatory mediators like TNFα and nitric oxide (15, 16). Doppler ultrasonography quantifies these hemodynamic changes, supporting assessment of inflammation and its severity (17, 18). Therefore, Doppler ultrasonography, which quantifies uterine blood flow (UBF), may detect inflammation-associated hyperemia and serve as a non-invasive adjunct for SCE detection (19). Doppler has been widely applied to monitor estrous cycles (20), pregnancy (21), placental separation (22), uterine involution (23), uterine torsion (24) and incomplete cervical dilatation (25). Pelvic inflammatory disease in women is associated with increased UBF and low resistance index (RI) and pulsatility index (PI) (26). Comparable patterns are reported in *Bos taurus* cattle (27, 28), buffaloes (19), mares (29), and ewes (30), where endometritis elevates perfusion and lowers Doppler indices. Despite these insights, systematic characterization of these hemodynamic shifts in *Bos indicus* cattle across the estrous cycle remains lacking. Addressing this gap is essential, as indigenous breeds like Tharparkar form the genetic and economic backbone of tropical livestock systems. Here, we employed transrectal Doppler ultrasonography throughout the estrous cycle in healthy and Tharparkar cows with SCE to ascertain the effect of SCE on uterine vascular perfusion. We hypothesized that SCE would be associated with distinct hemodynamic signatures in Doppler indices, blood flow velocity and volume, and arterial diameter with uterine hyperemia.

## Materials and methods

### Location and climatic parameters

The experiment was conducted at the Cattle and Buffalo Farm, ICAR–IVRI, Izatnagar, India (28° N, 79° E; 564 m above mean sea level). The climate of the region is humid and tropical, with a mean summer temperature of 30.70 °C (range 15.10–40.90 °C) and mean relative humidity of 53.20%.

### Animals and feed management

Twenty lactating parous Tharparkar cows (parity 2–3; age 4.80 ± 0.81 year; body weight 388.1 ± 9.5 kg; mean milk yield 3.01 ± 0.23 kg day^−1^) were enrolled. Provide days in milk (DIM) or postpartum interval for each animal (70 ± 3.34). Cows received 1.5–2.5 kg concentrate feed (20% DCP, 70% TDN), ad libitum wheat straw, green fodder twice daily, and free access to water. Animals were managed under semi-intensive conditions with access to open paddocks.

### Experimental design, inclusion and exclusion criteria of animal

**Figure 1:**
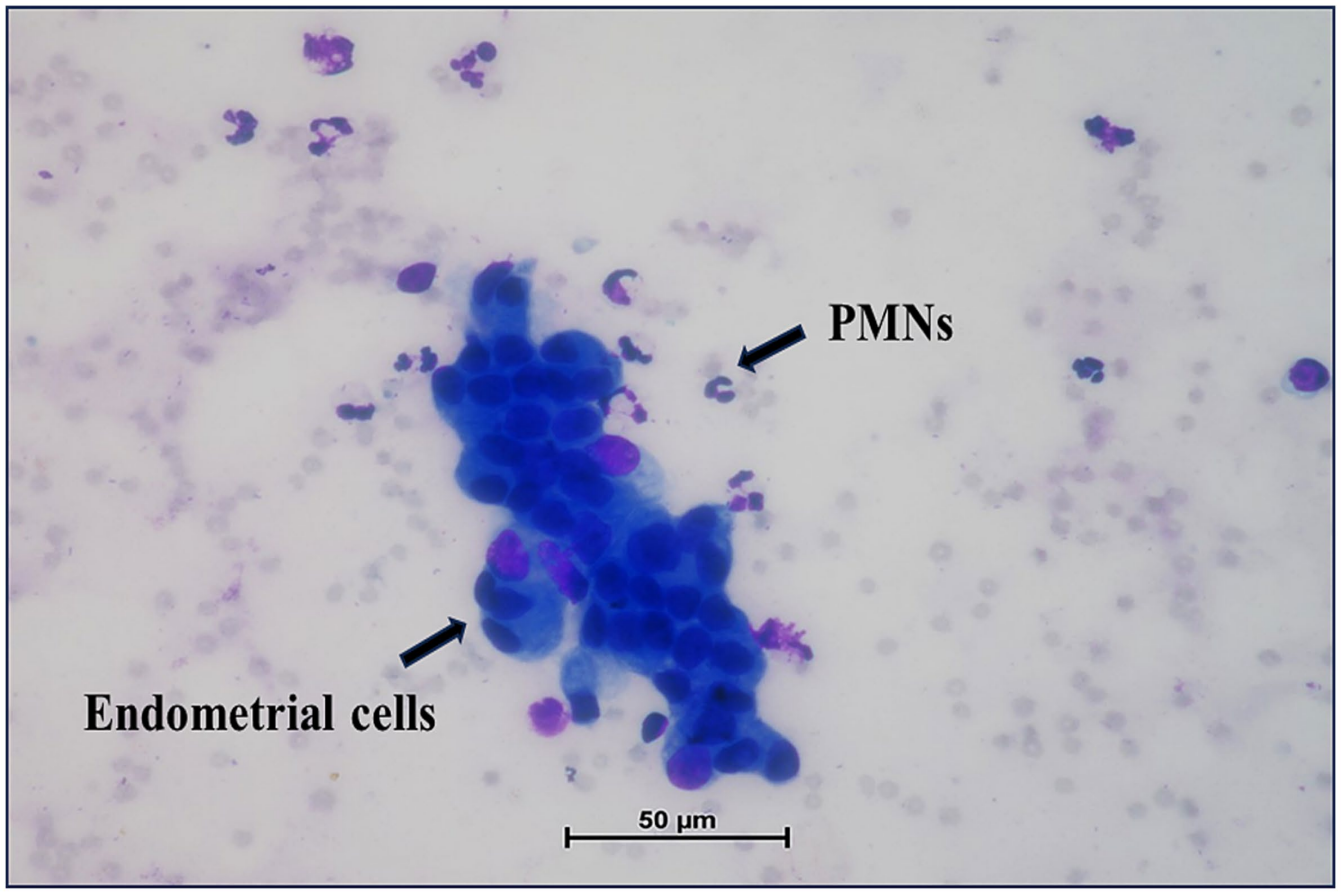
Endometrial cytology stained with Wright’s Giemsa stain (40×).

Before the start of the experiment, all animals underwent clinical screening to exclude systemic or inflammatory conditions. Reproductive tract evaluation included visual vulvar inspection and vaginal palpation; clear mucus indicated absence of clinical endometritis (31). Animals found positive for uterine infections were excluded from the study. Only clinically normal cows with regular estrous cycles, no history of uterine infection, dystocia, or retained placenta, and no abnormal discharge were included. Additionally, transrectal palpation and B-mode ultrasonography confirmed uterine integrity, with no intrauterine fluid or abnormalities. No hormonal synchronization of estrous cycles was performed. Each cow was enrolled on a random day of its cycle, and daily ultrasonographic monitoring was initiated to track the growth of the dominant follicle. Once natural behavioural estrus was detected, confirmed by the presence of a preovulatory follicle and clear mucous discharge, endometrial cytology sampling for SCE diagnosis was performed using the cytobrush technique (6). Subsequently, ultrasonographic monitoring continued throughout one complete estrous cycle at regular intervals to assess uterine haemodynamics under natural conditions without exogenous hormonal intervention. Smears were stained with modified Wright’s Giemsa, and 100 cells per slide were counted (Figure 1). Based on established cytological criteria, SCE was defined as the presence of ≥5% PMNs at ≥47 days postpartum (6). Ten cows with SCE formed the treatment group, and ten healthy cows served as controls. Post-cytobrush welfare monitoring was performed for 24 h using a standardized 5-point pain score and observation for abnormal discharge, as per ARRIVE 2.0 guidelines (32). No adverse reactions were noted. To minimize observer bias, the ultrasonography operator remained blinded to the group allocation (control or SCE-affected).

### Spectral Doppler acquisition

The MUA imaging was performed using a transrectal linear transducer (Exago ECM, France) with a frequency of 7.5 MHz using color and spectral Doppler. The abdominal aorta was traced caudally to the internal/external iliac bifurcation and the MUA localized within the mesometrium using color Doppler. Spectral Doppler was applied with the sample gate centered within the lumen and gate length set approximately to vessel diameter. The insonation angle was kept ≤60° (mean used: 50°), and color gain was standardized (~20 dB). Pulse repetition frequency (PRF) was adjusted (typically 3,000–5,000 Hz; in this study 4,000 Hz) to avoid aliasing; when aliasing occurred PRF or scale was adjusted and angle reconfirmed. For each MUA, recordings were accepted only when at least three consecutive, high-quality, consistent cardiac cycles were visualized; three such waveforms were frozen and used to calculate indices, and the mean of these three measurements per side was used for analysis (33, 34). Each session required ~30–45 min per cow and was performed every 3 days from estrus (D0) through the next estrus (D21).

### Operator training and quality control

A single, experienced operator performed all Doppler scans using a standardized protocol and machine presets to minimize observer-related variation. Weekly reliability tests demonstrated excellent reproducibility (intra-observer ICC = 0.92, inter-observer ICC = 0.88). All waveforms were quality-checked, and those with artifacts (e.g., motion, aliasing) were excluded and reacquired.

### Measurement of spectral Doppler attributes of the middle uterine artery

The RI and PI were calculated automatically by the ultrasound system using standard formulas (35):


RI=(PSV−EDV)/PSV

PI=(PSV−EDV)/mean velocity

Gosling and King (35) commonly used convention; see Ginther and Utt (17) for Doppler principles. Timed-averaged maximum velocity (TAMV; units cm s^−1^) was taken as the system’s time-averaged peak velocity across the cardiac cycle. Blood flow volume (BFV, mL min^−1^) was estimated using (36):


$$\mathrm{BFV}=\text{TAMV}\times \pi \times {\left(D/2\right)}^{2}\times 60,$$

where *D* is the internal vessel diameter (cm) measured intima-to-intima, and the factor 60 converts seconds to minutes. Reported BFV thus represents an approximation assuming laminar flow and circular cross-section; limitations of this calculation are acknowledged.

### Measurement of diameter of MUA

Internal vessel diameter (intima-to-intima) was measured on three cardiac-matched frozen B-mode frames at the Doppler sampling site; the mean of three measurements per side was used (34).

### Blood sampling and progesterone (P4) estimation

After each Doppler exam, 4 mL blood was collected by jugular venipuncture into serum clot-activator tubes, allowed to clot for 30 min at room temperature, and centrifuged at 3,000 rpm for 10 min. Serum was aliquoted and stored at −20 °C. Progesterone (P4) was measured by DRG progesterone ELISA (EIA-1561); assay sensitivity = 0.045 ng mL^−1^, intra-assay CV = 6.4%, inter-assay CV = 6.6%. Samples were analyzed in duplicate.

### Statistical analysis

Data were analyzed using GraphPad Prism 10.6.0 (GraphPad Software, LLC, San Diego, CA, United States) following standard methods. Normality and distribution of data were determined using Shapiro–Wilk and Kolmogorov–Smirnov normality test. The mean values of serum P4 concentration, Doppler attributes of MUA viz., Doppler indices (RI, PI), TAMV, vessel diameter and BFV were tested for significance using independent *t*-test. The RI, PI, TAMV, diameter of MUA and BFV were subjected for analysis of variance of repeated measurement considering the variance between animals as well as between days of estrous cycle, using GLM procedure to determine the principal effects and their interaction. The statistical model was *y_ij_* = *μ* + *α_i_* + *τ_j_* + (*ατ*)*_ij_* + ϵ *_ij_*, where *α_i_* is the day effect and *τ_j_* is the animal effect and (*ατ*)*_ij_* is their interaction effect. The significance of the differences was tested using *post-hoc* Sidak test. The association among Doppler indices, TAMV, BFV, Cl size, and serum P4 concentration were calculated using Pearson’s correlation coefficient. All the data presented are as mean ± standard error of mean. The difference in mean values for all data analyzed with *p* < 0.05 was considered significant.

## Result

### PMNs count in endometrial cytology screening

Ten cows classified as SCE-positive had a mean endometrial PMN% of 6.80 ± 0.24%, whereas the control group had mean PMN% = 3.30 ± 0.21% (group difference *p* < 0.0001).

### Spectral Doppler attributes of MUA

**Figure 2:**
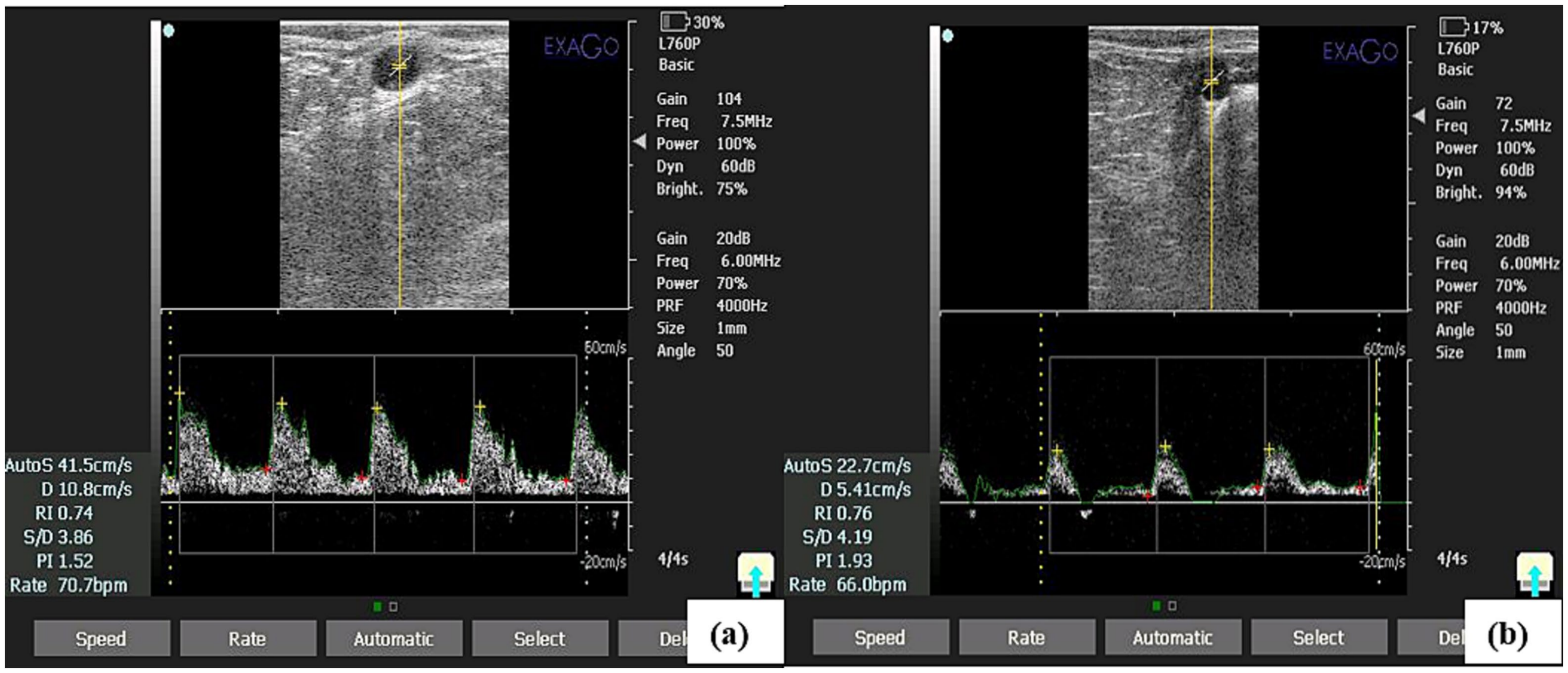
Representative spectral Doppler sonograms showing the variation of blood flow measured from the middle uterine artery on **(a)** follicular phase **(b)** luteal phase in SCE affected Tharparkar cows.

**Figure 3:**
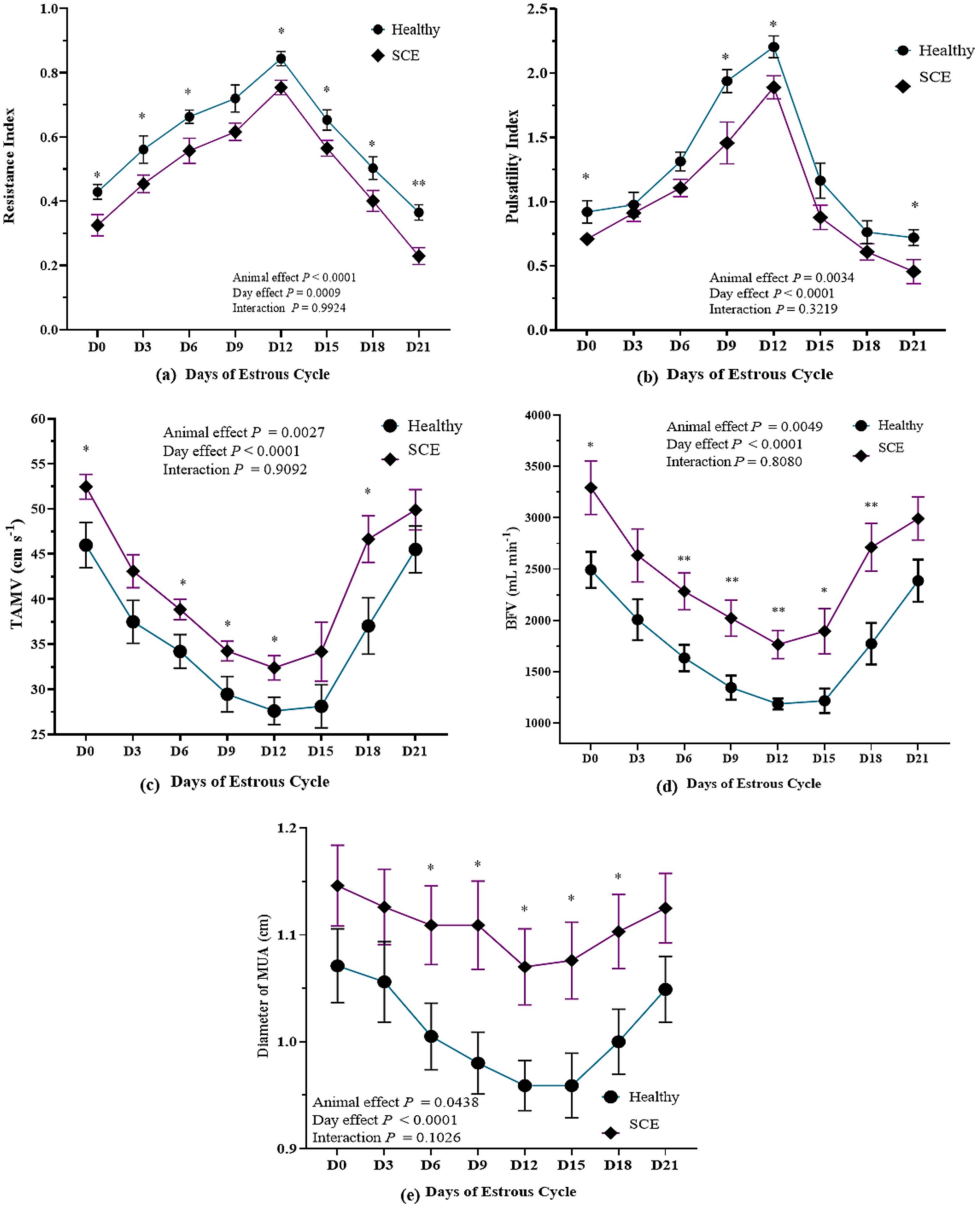
Spectral Doppler attributes **(a)** resistance index (RI), **(b)** pulsatility index (PI), **(c)** timed-averaged maximum velocity (TAMV), **(d)** blood flow volume (BFV), and **(e)** diameter of middle uterine artery (MUA) of healthy and subclinical endometritis affected Tharparkar cows. Value with asterisks (*) mark the significant differences.

**Figure 4:**
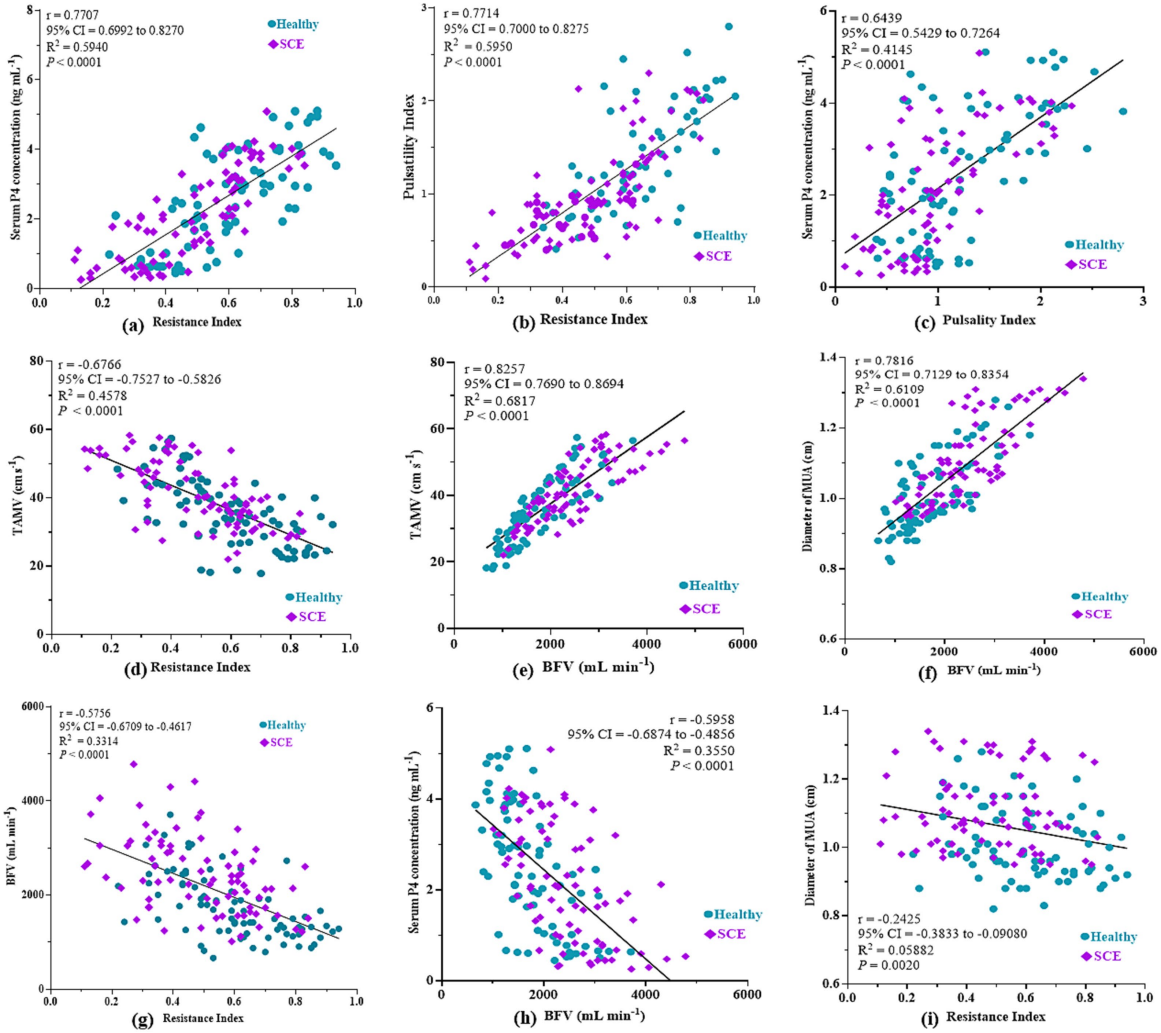
Correlation analyses among Doppler indices, time-averaged maximum velocity (TAMV), blood flow volume (BFV), diameter of the middle uterine artery (MUA), and serum P_4_ concentration in Tharparkar cows with and without subclinical endometritis. Panel **(a)** shows the correlation between resistance index (RI) and serum P_4_ concentration; **(b)** RI and pulsatility index (PI); **(c)** PI and serum P_4_ concentration; **(d)** RI and TAMV; **(e)** BFV and TAMV; **(f)** BFV and MUA diameter; **(g)** RI and BFV; **(h)** BFV and serum P_4_ concentration; and **(i)** RI and MUA diameter.

Mean RI (average of left and right MUA) was significantly lower in SCE cows than in controls at ovulation (day 0), early luteal (days 3, 6), mid-luteal (days 9, 12), late luteal (days 15, 18) and at the subsequent ovulation (day 21) (*p* < 0.05), indicating increased uterine perfusion (Figures 2a,b, and 3a). RI was significantly influenced by both animal status and day of the estrous cycle (*p* < 0.05), while their interaction was non-significant (*p* > 0.05). RI showed positive correlations with CL size (*r* = 0.64; *p* < 0.0001) and serum P4 concentration (*r* = 0.77; *p* < 0.0001) (Figure 4a), and exhibited a coefficient of variation of 25.54%. PI demonstrated a similar trend, being significantly lower in SCE cows at ovulation (day 0), days 9 and 12, and at the subsequent ovulation (day 21) (*p* < 0.05) (Figures 2a,b, and 3b), with significant effects of animal status and cycle day (*p* < 0.05) but a non-significant interaction (*p* > 0.05). RI and PI were positively correlated (*r* = 0.77; *p* < 0.0001) (Figure 4b), while PI was negatively correlated with serum P4 (*r* = 0.64; *p* < 0.0001) (Figure 4c). The coefficient of variation for PI was 50.37%. Mean TAMV (average of right and left MUA) was higher in SCE cows (*p* < 0.05), with significant effects of animal status and cycle day, but without a significant interaction (*p* > 0.05) (Figure 3c). TAMV was negatively correlated with RI (*r* = −0.67; *p* < 0.0001) (Figure 4d). BFV was also higher in SCE-affected cows on days 0, 6, 9, 12, 15 and 18 of the estrous cycle (*p* < 0.05), with significant main effects of animal status and cycle day but no interaction (*p* > 0.05) (Figure 3d). BFV correlated positively with TAMV (*r* = 0.82; *p* < 0.0001) (Figure 4e) and MUA diameter (*r* = 0.78; *p* < 0.0001) (Figure 4f), and negatively with RI (*r* = −0.57; *p* < 0.0001) (Figure 4g) and serum P4 (*r* = −0.59; *p* < 0.0001) (Figure 4h). MUA diameter was consistently greater in SCE cows, with significant differences on days 6, 9, 12, 15 and 18 (*p* < 0.05). Diameter was significantly influenced by animal and cycle day (*p* < 0.05), with a non-significant interaction (*p* > 0.05) (Figure 3e). A weak negative correlation was observed between MUA diameter and RI (*r* = −0.24; *p* < 0.0001), indicating a minor trend toward higher resistance in smaller vessels (Figure 4i).

### Effect of production traits on blood flow indices

This study enrolled all cows were of similar physiological and management status, maintained under uniform feeding, housing, and milking schedules. Thus, effect of production traits (milk yield, parity, or days in milk were non-significant (*p* > 0.05) uterine haemodynamics indices (RI and PI) in either group, indicating physiological uniformity among experimental animals. All cows were maintained under similar nutritional and management conditions, minimizing the likelihood of production-related variation. Hence, the observed alterations in UBF can be primarily attributed to subclinical endometritic status rather than differences in production performance.

## Discussion

This study demonstrates that SCE in Tharparkar cows is associated with distinct uterine hemodynamic changes, measurable by Doppler ultrasonography across the estrous cycle. In our findings, both the RI and PI were significantly lower throughout the estrous cycle in cows with SCE. Our results are consistent with reports of reduced RI and PI in buffalo (19) *Bos taurus* cattle with uterine infection (27, 28, 37) and in women with pelvic inflammatory disease (26). Furthermore, the association between UBF changes and severity of pelvic inflammatory disease, whether acute or resolved, has been well-documented (38–40). The convergence of evidence across species supports the concept that uterine inflammation produces a conserved hemodynamic signature, that is hyperemia with low RI and PI. Additionally, in both groups, the lower RI and PI values observed during the follicular phase indicate increased UBF to the reproductive organs, a pattern consistent with previous findings (34). Uterine blood flow increases during estrus (41), and lower RI/PI during the follicular phase has been previously reported (33). This follicular rise in perfusion is likely mediated by estradiol-induced vasodilation and associated uterine changes (42, 43). Mechanistically, estrogen may alter vascular tone via reduced calcium influx in smooth muscle or by up-regulating endothelial nitric oxide synthase (eNOS), increasing NO availability and vasodilation (44, 45). Our finding of a strong RI–PI correlation is consistent with past Doppler studies (28, 34). Moreover, we observed a positive correlation of RI with PI, CL size, and serum P4 also between PI and serum P4 corroborating previous studies (28, 34). The TAMV and BFV were significantly higher in Tharparkar cows affected by SCE compared to healthy cows, consistent with previous findings (19, 28, 37). Additionally, in support of our findings an increased UBF associated with endometrial inflammation in mares (29) and acute uterine inflammation in ewe (46). Increased BFV and TAMV could be mediated by nitric oxide (NO), a potent vasodilator; previous work reports higher NO levels in uterine secretions and blood in cattle with SCE (1.6 times) and clinical endometritis (2.2 times) (47). We did not measure NO here, so future studies combining biochemical markers (e.g., NO metabolites, cytokines) with Doppler measures are warranted. Furthermore, an inverse relationship between TAMV and BFV with RI, BFV with serum P4 and positive correlation of BFV with diameter of MUA and TAMV were also confirmed corroborating previous findings (33, 34, 36). In this study, we critically evaluated the influence of uterine inflammation on the diameter of the MUA as an important measure of UBF perfusion. Our findings indicated that the diameter of the MUA was significantly greater in cows with SCE, which is in alignment where an increased diameter of the uterine artery in cattle (28, 37) and buffaloes (19) with uterine infection were reported. In contrast, the diameter of the uterine artery does not significantly correlate with increased UBF in induced endometritis (27, 48, 49), suggesting variability in findings across different studies. Furthermore, we observed an inverse relationship between the diameter of the MUA and RI values, supported by earlier findings (28, 36). This inverse correlation indicates hyperaemia of uterine tissue in cows with SCE, associated with increased diameter of the middle uterine artery. A limitation of this study is the modest sample size (*n =* 10/group), which reduces power for detecting interactions and may restrict generalizability. Furthermore, we did not perform concurrent bacteriological assessments or measure systemic inflammatory markers such as serum amyloid A or haptoglobin, nor did we directly quantify nitric oxide. Our findings therefore apply specifically to lactating Tharparkar cows and require confirmation in larger, independent populations.

In conclusion, SCE in Tharparkar cows produces consistent alterations in uterine artery blood flow, detectable by Doppler ultrasonography. These hemodynamic shifts represent initial Doppler reference values for an indigenous *Bos indicus* breed, highlighting that uterine inflammation leaves a quantifiable vascular signature. Future integration of Doppler indices with biochemical markers such as nitric oxide may enhance diagnostic accuracy and support timely intervention strategies. With validation in broader populations, Doppler metrics could serve as a non-invasive adjunct for detecting uterine inflammation and guiding reproductive management in tropical dairy systems.

## Data Availability

The raw data supporting the conclusions of this article will be made available by the authors, without undue reservation.
